# DNA-Decorated PET Nanochannels for Sensitive Biosensing

**DOI:** 10.3390/bios15110751

**Published:** 2025-11-10

**Authors:** Xianyan Gong, Hongquan Xu, Xigui Zhang, Dagui Wang

**Affiliations:** 1 Yangtze Delta Region Institute (Huzhou), University of Electronic Science and Technology of China, Huzhou 313001, China; 2 Faculty of Materials Science and Chemistry, China University of Geosciences, Wuhan 430070, China

**Keywords:** porous materials, functional, sensors, thin films, ion transport

## Abstract

Functionalized nanochannels are crucial for achieving excellent ion transport properties and enable versatile applications such as ion gating, biosensing, and energy conversion. Conical single nanochannels were fabricated in single-ion-track polyethylene terephthalate (PET) membranes using the ion-track-etching method. Leveraging the high programmability of deoxyribonucleic acid (DNA) strands, a series of DNA molecules were designed to functionalize the outer surface at the tip region (small opening) of the conical PET nanochannels. This approach enabled precise regulation of both spatial charge distribution and steric hindrance on the outer surface, enabling the investigation of ion transport properties under the dominance of outer surface charge effects across ions of different valences. In contrast to the low-valence K^+^, the high-valence cation Ru(NH_3_)_6_^3+^ exhibited far greater enhancement in ionic current rectification (ICR) within PET films functionalized with DNA of varying charge densities. We used COMSOL simulations to corroborate that higher-valence ions exert more pronounced effects on ion transport in conical nanochannels with greater outer surface charge density. Furthermore, it was confirmed that the tip region plays a critical role in modulating the ion transport properties of conical nanochannels, thereby validating outer surface functionalization as a rational and efficient strategy.

## 1. Introduction

Inspired by diverse ionic nanochannels in biological systems, functionalized solid-state nanochannels fabricated via nanotechnology exhibit outstanding ion transport properties comparable to their biological counterparts [1,2,3,4]. Therefore, scientists have developed a series of biomimetic solid-state nanochannels by emulating the properties of biological nanochannels. The functionality of synthetic solid-state nanopores and nanochannels is inherently constrained by the substrate materials used in their fabrication [5,6]. Consequently, functionalization serves as the primary approach to enrich the capabilities of these biomimetic pores and channels. Modification strategies for solid-state nanochannels are generally classified into two categories: symmetric and asymmetric modifications, with assembly methods varying according to the properties of the functional molecules. Symmetric modification involves uniform functionalization of the entire inner surface of nanochannels. This relatively straightforward approach alters the overall physicochemical properties of nanochannels through molecular modification. For instance, nanochannels with intrinsic functional groups (e.g., carboxyl groups) can be directly chemically modified in solution via amide bonding for diverse molecular functionalization [7,8,9,10]. Alternatively, electrostatic self-assembly methods have been employed to modify nanochannels with excessive surface charges [11,12,13,14]. Therefore, asymmetric modification is predominantly applied to nanochannels rather than nanopores, owing to their higher depth-to-diameter ratio. This high aspect ratio is essential for establishing concentration gradients of functional molecules within the channels, enabling asymmetric functionalization of the inner surface. Reported asymmetric modification techniques include ion sputtering [15,16], asymmetric solution chemical modification [17,18,19], physical/chemical vapor deposition [20,21], plasma-induced modification [22,23,24], and asymmetric self-assembly methods [25,26].

These artificial nanochannels have found applications in diverse fields such as biosensing, ion gating, energy conversion, and nanofluidic devices [27,28,29,30]. In the biosensing field, Martin et al. first reported the integration of DNA into solid-state nanochannels to construct artificial ion channels [31]. Subsequently, Guo et al. designed a DNA “super-sandwich” self-assembled structure that enhanced the sensitivity of nanopores to oligonucleotide-specific sequences [32]. In this work, these target DNA-blocked nanopores could be reopened through the addition of adenosine triphosphate (ATP) molecules. However, in conventional studies of functionalized solid-state nanochannels, functional molecules are typically randomly distributed throughout the entire nanochannel structure. Notably, in the aforementioned research applications, functionalization has primarily focused on the inner surface of nanochannels. To further enhance the performance of solid-state nanochannels, researchers have focused on precise functionalization strategies that involve distinct spatial partitioning, targeting the inner and outer surfaces separately [33,34]. Li et al. achieved controlled deposition of gold or titanium on porous anodic aluminum oxide (AAO) membranes via electron beam evaporation, creating distinct hierarchical layers inside and outside the nanochannels implementing three precise regional modification strategies: outer-surface functionalization, inner-surface functionalization, and dual inner–outer surface functionalization [35]. The versatility of nanopore sensing can be further enhanced by incorporating programmable molecular mechanisms. In particular, DNA hybridization and conformational changes have emerged as powerful strategies for designing highly specific and responsive sensors [36,37,38]. Previous studies have demonstrated that the sensitivity of ion transport within nanochannels is critically dependent on surface charge effects [39,40]. However, existing research has predominantly concentrated on the influence of the inner surface on ion transport performance, often overlooking the significant role of the outer surface [41]. The relationship between the spatial charge distribution and multivalent ions at the outer surface has not been systematically investigated.

Based on the above analysis, we functionalized the outer surface of conical nanochannels with programmable DNA molecules to modulate the spatial charge distribution, thereby altering their asymmetric ion transport properties. In this work, conical nanochannel PET membranes were fabricated using the ion-track-etching method. Leveraging the programmable nature of DNA, the outer surface of the nanochannels was functionalized by grafting DNA strands with varying charge densities, enabling precise control over the spatial charge distribution on the outer surfaces. The functionalized PET nanochannels modified with long-strand DNA rich in negative charges (RCA-DNA) exhibited excellent sensitivity in the selectivity and transmission of high-valence cations. Furthermore, capitalizing on the phenomenon in which multivalent ions alter the charge distribution and consequently affect the ion selectivity of nanochannels, this study systematically investigates the influence of ion valence states on asymmetric ion transport behavior governed by the outer surface charge effects. COMSOL simulations further demonstrate that the surface charge density on the outer surface of conical single nanochannel significantly influences the transport of high-valence cations.

## 2. Experimental Details

Preparation of PET film: Nanochannels were prepared via ion track etching; the equipment is shown in Figure S1. In this work, we set specific experimental conditions as follows: (a) Each PET film side was irradiated with UV light (365 nm, 1.0 mW cm^−2^) for 1 h. (b) The PET membrane treated in step (a) was then secured at the center of the polytetrafluoroethylene electrochemical test groove. (c) A 9 M NaOH etching solution and 1 M KCl + 1 M HCOOH corrosion inhibitor solution were, respectively, added to each side of the film in the test groove and heated in a 35 °C water bath. (d) A voltage of 1 V DC was applied to a side of the etching solution for 10 min, and then the etched channel was connected and the PET film was immersed in the corrosion inhibitor solution for 30 min. (e) The above PET films were washed with deionized water for 10 times, left in deionized water for more than 12 h, and then dried with N_2_ gas.

DNA functionalization of outer surface: Firstly, a clean PET film was disposed with plasma treatment in argon atmosphere for 10 min (plated with 2 nm Cr and 2 nm Au). Secondly, a series of different sequences of DNA molecular chain solutions (A-3, Poly-A, Poly-A+T, RCA-DNA) (see Table S1) were dripped on the side of a small hole of the conical nanochannel PET film for 1 h. Poly-A is easy to adsorb on gold surfaces; thus, it was grafted on the outer surface of the nanochannel [42,43]. Thirdly, the above PET film was cleaned with the residual solution and dried with N_2_ gas.

Characterization: The surface morphology and element composition of the PET film were characterized using field emission scanning electron microscopy (FESEM, SU8010, Hitachi, Tokyo, Japan) with an energy-dispersive spectrometer (EDS). The element distributions along the depth of the functional nanochannels were measured using X-ray photoelectron spectroscopy (XPS) (ESCALAB Xi, Thermo Fisher Scientific, Waltham, MA, USA) and a time-of-flight secondary ion mass spectrometry (ToF-SIMS) (IONTOF, GmbH, Münster, Germany). The Zeta potential of the PET film was characterized with an electrokinetic analyzer for solid samples (Surpass ANTON PAAR, Graz, Austria).

## 3. Results and Discussion

**Figure 1:**
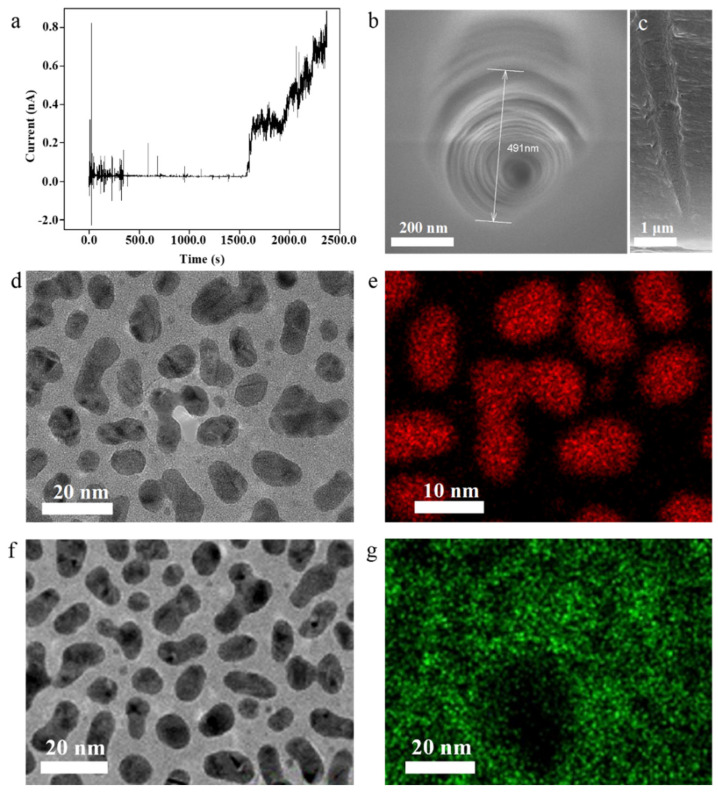
(**a**) Current–time curve of conical nanochannel etching process; FESEM of conical nanochannel surface (**b**) and cross-section (**c**); high-resolution TEM image (**d**) and EDS mapping (**e**) of Au NPs; high-resolution TEM image (**f**) and EDS mapping (**g**) of Cr NPs.

We added a constant voltage of 1 V into the etching process of the conical nanochannel for the driving motion of the etching ions to obtain the desired shape. The etching condition was known by observing the current change at any one timepoint, and the nanochannel had formed at 1600 s due to significant current increase (Figure 1a). The aperture gradually increased with increasing etch time. A conical nanochannel with a large pore end of about 500 nm and a length of 4.9 μm was prepared (Figure 1b). A cross-sectional SEM image clearly shows the etched conical nanochannels (Figure 1c). Electrochemical characterization of the single etched conical nanochannel was performed using 1 M KCl solution, yielding the current–voltage curve presented in Figure S2. The high concentration of the KCl solution resulted in a compressed electrical double layer within the nanochannel, leading to diminished current rectification that was utilized to determine the tip diameter. Based on the characterized large-pore-end diameter and the current curve mentioned above, the values were substituted into Equation (1) [44]. The calculated small-pore-end size was approximately 9.5 nm. Therefore, the conical nanochannel etched had a large pore end of about 500 nm and a small pore end of about 9.5 nm.

(1) d=4LI/πDκU

where d is the size of the small pore end, L is the length of the nanochannel, I/U is the current–voltage ratio measured in 1 M KCl solution, D is the current at the large hole end, π is Pi, and κ is the solution conductivity (at 25 °C, the conductivity of the 1 M KCl solution is 0.11173 Ω^−1^·cm^−1^). To facilitate the functionalization of nucleic acid molecules on the outer surface of the pore and further reduce the effective aperture, the outer pore surface was coated with a Au NP layer. A chromium layer beneath the gold serves as an adhesive interlayer, enhancing the stability of the Au NP coating on the pore surface. Figure 1d,e show that the outer surface of the conical nanochannel was treated with Au NP spraying in order to better modify DNA on the outer surface. Cr element coating acts as an adhesive to make the Au NPs more stable on the outer surface (Figure 1f,g).

**Figure 2:**
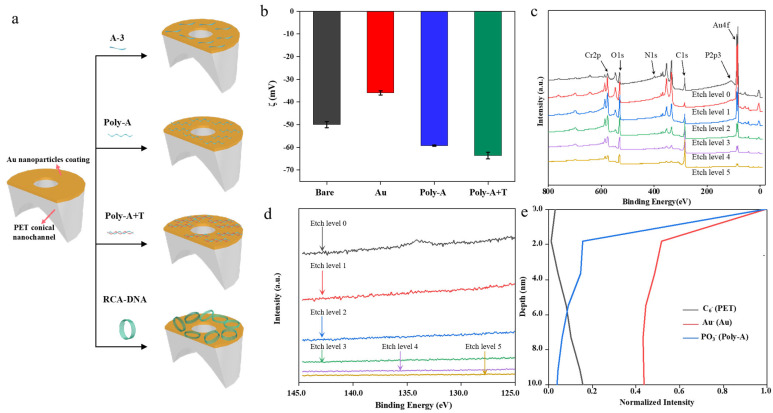
(**a**) Schematic of DNA modified on PET film surfaces; (**b**) solid-state Zeta potential; (**c**) XPS full spectrum and (**d**) XPS high-resolution spectrum of N element; (**e**) ToF-SIMS image of PET film modified with DNA. Three experimental replicates were performed on three different substrates of the same kind for each error bar.

A series of different sequences of DNA molecular chain solutions (A-3, Poly-A, Poly-A+T, RCA-DNA) were modified on the side of a small hole of the conical nanochannel PET film (Figure 2a). To characterize the functionalization of nucleic acid molecules on the outer surface, solid-state Zeta potential measurements were employed to analyze the outer surface of the PET nanochannel. We characterized the surface charge density of the PET film modified with DNA with the Zeta potential, as shown in Figure 2b. The negative Zeta potential on the outer surface of the conical nanochannel gradually increases because both the PET film and DNA are negatively charged. This changed the charge density distribution on the outer surface of the conical nanochannel. To further provide direct visual evidence that the modified DNA is distributed on the outer pore surface, this study conducted XPS depth profiling on the pore structure. Characterizing the distribution of phosphorus (P) from the DNA demonstrated that the DNA modification was located exclusively on the outer surface. The peak of elements P (a DNA-specific element) and Au in the XPS spectrum were observed on the outer surface and disappeared as the etching depth increased, as shown in Figure 2c,d. To precisely characterize the thickness of the DNA layer on the outer surface of the nanochannels, this study employed ToF-SIMS to determine the chemical stoichiometry of various elements on the nanochannel’s outer surface. The DNA was only grafted on the outer surface and edge of the nanochannel, and DNA rarely entered the nanochannel. We determined the stoichiometry of each component on the outer surface of the conical nanochannel (within 10 nm) via ToF-SIMS characterization (Figure 2e). The strong signal of PO_3_^−^ (DNA, Poly-A) and Au^−^ (Au NPs) at 0–3 nm and rare signal after 3 nm indicate that the thickness of the DNA modified on the outer surface of the conical nanochannel was less than 3 nm. This is consistent with the thickness of one base molecule tiled on the surface. The DNA did not enter the conical nanochannel because the signal of C_6_^−^ (PET film) increased, while the signal of PO_3_^−^ disappeared.

**Figure 3:**
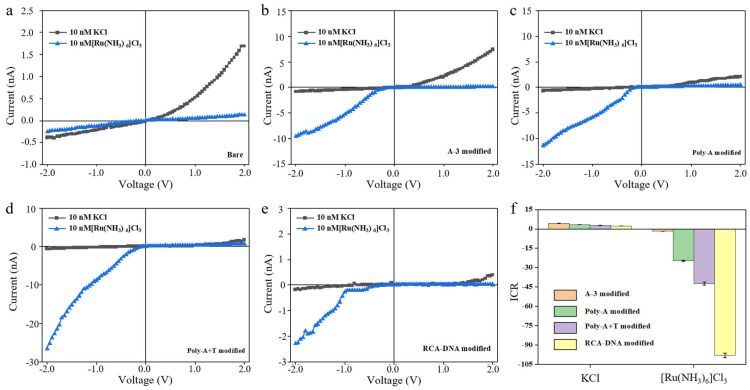
Current–voltage diagram of outer surface of conical nanochannels modified with a series of DNA: (**a**) bare, (**b**) A-3, (**c**) poly-A, (**d**) poly-A+T, and (**e**) RCA-DNA. (**f**) ICR comparison diagram of outer surface of conical nanochannels with different charges, with three experimental replicates performed on three different substrates of the same kind for each error bar.

Regarding the properties of the outer surfaces of the pores determined using different assembly and disassembly strategies, electrochemical tests were conducted on the pore, and the current–voltage curve obtained is shown in Figure 3. The concentration of the test solution in the figure is 10 mM, and the aperture of the conical pores remains consistent. Figure 3a shows the current of the bare conical nanochannel PET film under test (the ground end is the large hole end, and the potential end is the small hole end). There is a small negative current and a large positive current due to the bare PET film itself having a negative surface charge. When multivalent ion solutions pass through the nanopore, a clear reversal of the preferential direction of ion current rectification is observed, along with an overall increase in current magnitude. This phenomenon occurs because positively charged ions are attracted to the negatively charged inner wall of the pore, and they adsorb onto the surface. This process alters the overall surface charge properties of the pore, transforming it from negatively charged to positively charged, thereby leading to the reversal of the rectification direction. The current signal of the functional conical nanochannel modified with DNA of different charge densities (A-3, Poly-A, Poly-A+T, RCA-DNA) shows an obvious ICR phenomenon due to the change in the surface charge density (Figure 3b–e). As shown in Figure 3b,c, both short-chain DNA (A-3) and Poly-A modifications led to a further increase in current magnitude and more evident rectification reversal in the presence of Ru(NH_3_)_6_^3+^, while the current in the standard KCl solution remained largely unchanged. To further enhance the outer surface charge density, Poly-A+T was formed via DNA complementary self-assembly and grafted onto the outer surface. The corresponding I-V curve (Figure 3d) shows a relative increase in current and enhanced rectification reversal. These results indicate that higher outer surface charge density strengthens charge effects and amplifies the influence of ion valence on nanochannel ion transport. After confirming the role of ion valence in modulating transport via outer surface charge, we further increased the charge density using rolling circle amplification (RCA) to generate a long cyclic DNA structure. This introduced high-density DNA onto the outer surface, significantly enhancing both surface charge and steric hindrance. As shown in Figure 3e, while the current in KCl remained similar, a clear rectification reversal was observed for Ru(NH_3_)_6_^3+^.

To further quantify the ionic rectification phenomenon, the ion current rectification (ICR) ratio was calculated. The ICR ratio was calculated in order to quantify the ICR phenomenon and is defined in two ways: (1) *I*_+2_/*I*_−2_ > 1, ICR = |*I*_+2_/*I*_−2_| and (2) *I*_+2_/*I*_−2_ < 1, ICR = *I*_−2_/*I*_+2_. Positive and negative ICR ratios represent different dominant potential directions of ICR. Figure 3f shows the ICR ratio of the conical nanochannel PET film modified with four different sequences of DNA. Compared to single-stranded Poly-A and double-stranded Poly-A+T, the ion aggregation effect at the tip of the conical nanochannel was more significant with the negative charge density increasing on the outer surface, for which the ion transport barrier was improved and ICR ratio was increased. Since PET is negatively charged and the DNA molecules used for modification are also negatively charged, the negative charge on the outer surface gradually increases as the amount of DNA modification rises. This enhances the attraction to positively charged ions, making the ion selectivity change induced by multivalent cations more significant. This is clearly reflected in the greatly enhanced ICR ratio for Ru(NH_3_)_6_^3+^ in RCA-DNA modified channels, where rectification reversal was most substantial, indicating a stronger effect of high-valence ions in conical nanochannels with highly charged outer surfaces.

**Figure 4:**
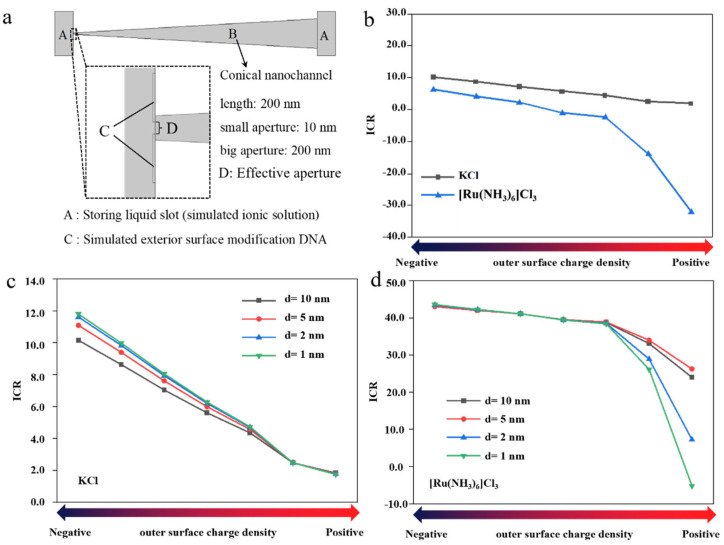
(**a**) COSMOL simulation model and local enlarged view of conical single nanochannel, (**b**) ICR vs. outer surface charge density. Simulating the ion transport performance under spatial steric hindrance variations for different ionic solutions: (**c**) KCl, (**d**) [Ru(NH_3_)_6_]Cl_3_.

Ion transport experiments revealed that modifying the outer surface with negatively charged functional molecules further augmented the cation selectivity of the conical nanochannels, resulting in a marked increase in the rectification ratio for multivalent cation transport. However, due to limitations in the fabrication process of single conical nanochannels, the stability of the experimental results was relatively poor. To further validate the effect of the altered spatial charge distribution on the outer surface on the ion selectivity of conical nanochannels, this study employed simulation methods for investigation. By simulating the influence of the spatial charge distribution on ion transport within the channels and comparing the results with experimental data, the reliability of the experimental observations is enhanced, enabling further exploration of ion transport mechanisms. Figure 4 shows that the influence of the surface charge density and steric hindrance on ion transmission in the nanochannel was simulated by COMSOL. The base model for the COMSOL simulation and local enlarged view of the conical single nanochannel are shown in Figure 4a,b. (A) presents the reservoir, which simulates the ion solution and applied potential regions at both ends of the nanochannel. (B) is the conical nanochannel, with a length of 2000 nm, consistent with the experimental values—a small pore end simulated as 10 nm, and a large pore end simulated as 200 nm. (C) is a 1 nm thick rectangular block set on the outer surface of the small pore end, simulating the functional modification with DNA. By varying the distance and charge amount of the rectangular block, the effects of outer surface modification with functional molecules on the spatial steric hindrance and spatial charge distribution at the small pore end are simulated. The distance of the rectangular block is shown in (D), which represents the compression of the effective aperture d by the outer surface functional molecules. The electronegative nanochannel has a positive ICR ratio for the transmission of K^+^ and Ru(NH_3_)_6_^3+^, indicating that the dominant current direction is positive. The positive nanochannel has a significantly improved ICR ratio for high-valence cations (Ru(NH_3_)_6_^3+^). The higher charge density of the nanochannel has a significant effect on the transmission of high-valence ions, for which the ICR ratio is more obvious (Figure 4b). Meanwhile, we investigated the effect of steric hindrance changes by adjusting the effective aperture (d) on ion transmission (Figure 4c,d). Analysis of the three outer surface charge states (positive, neutral, and negative) reveals that steric hindrance alterations exert minimal influence on ion transmission (Figures S3–S5). The space charge density on the outer surface dominates the ion transport performance of the conical nanochannels, particularly for multivalent ions. Therefore, the conical PET nanochannel effect on the high-valence action ions can be regarded as changing the surface charge density on the outer surface of the PET nanochannel.

## 4. Conclusions

In this study, we achieved precise functionalization of the outer surface of conical nanochannels in PET films with strategically designed DNA molecules to modulate their surface charge density. Systematic investigations revealed that PET films modified with DNA of varying charge densities exhibited a significantly enhanced ionic current rectification (ICR) ratio for the high-valence cation Ru(NH_3_)_6_^3+^ compared to the low-valence cation K^+^. This phenomenon is attributed to the strong electrostatic interaction between the multivalent cations and the negatively charged DNA layer, which amplifies asymmetric ion transport behavior. The experimental findings were rigorously validated by COMSOL Multiphysics simulations, which confirmed that both the ion current and ICR ratio for high-valence cations are substantially augmented as the surface charge density on the nanochannel increases. Encouraged by the success of this surface-charge-mediated strategy for enhancing cation selectivity, we anticipate that such rationally designed nanochannels will pave the way for the development of more sensitive and selective platforms. By exploiting the programmable nature of DNA, such functionalized nanochannels could be adapted to detect a wide range of multivalent ionic species and biomolecules, including metal ions, proteins, nucleic acids, and small-molecule biomarkers, with improved sensitivity and specificity. Furthermore, the integration of functional nucleic acids such as aptamers or DNAzymes on the conical nanochannels of PET films could enable dynamic, responsive sensing systems for potential applications in synthetic biology and point-of-care testing.

## Data Availability

No data was used for the research described in the article.

## Supplementary Materials

The following supporting information can be downloaded at: https://www.mdpi.com/article/10.3390/bios15110751/s1, Figure S1: Schematic diagram of ion-track-etching device; Figure S2: The voltage-current curve of 1 M KCl solution test; Figure S3: The ICR ratio of the outer surface with positive charge nanochannels with a series of steric hindrance alterations (effecrive aperture, d = 1 nm, 2 nm, 5 nm, 10 nm); Figure S4: The ICR ratio of the outer surface with neutral charge nanochannels with a series of steric hindrance alterations (effecrive aperture, d = 1 nm, 2 nm, 5 nm, 10 nm); Figure S5. The ICR ratio of the outer surface with negative charge nanochannels with a series of steric hindrance alterations (effecrive aperture, d = 1 nm, 2 nm, 5 nm, 10 nm); Table S1: DNA sequences using in this work.
