# Supplementary material for: DNA-Decorated PET Nanochannels for Sensitive Biosensing
Source: Biosensors (Basel). 2025 Nov 10;15(11):751. doi: 10.3390/bios15110751 (PMC12650445; doi:10.3390/bios15110751)
Supplement: Supplementary file 1 [file biosensors-15-00751-s001.zip › biosensors-3962639-supplementary.pdf]

biosensors

Supplementary Materials

DNA-Decorated PET Nanochannels for Sensitive Biosensing

Xianyan Gong [1,2], Hongquan Xu [2], Xigui Zhang [1] and Dagui Wang [1,2,*]

1 Yangtze Delta Region Institute (Huzhou), University of Electronic Science and Technology of China, Huzhou 313001, China
2 Faculty of Materials Science and Chemistry, China University of Geosciences, Wuhan 430070, China
* Correspondence: wangdagui@uestc.edu.cn (D.W.); Tel.: +86-13260646383

Table S1. DNA sequences using in this work.

**Name**	**Length**	**Sequence（5′-3′）**
A-3	3	AAA
Poly-T	68	TTTTTTTTTTTTTTTTTTTTTTTTTTTTTTTTTTTTTTTTTTTTTTTTTTTTTTTT TTTTTTTTTTTT
Poly-A	68	AAAAAAAAAAAAAAAAAAAAAAAAAAAAAAAAAAAAAAAAAAAA AAAAAAAAAAAAAAAAAAAAAAAA
target DNA	12	TGTCTCGTTGCC
Linear DNA	60	GAGACATTTTTTTTTTTTTTTTTTTTTTTTTTTTTTTTTTTTTTTTTTTTTTTTTTG GCAAC

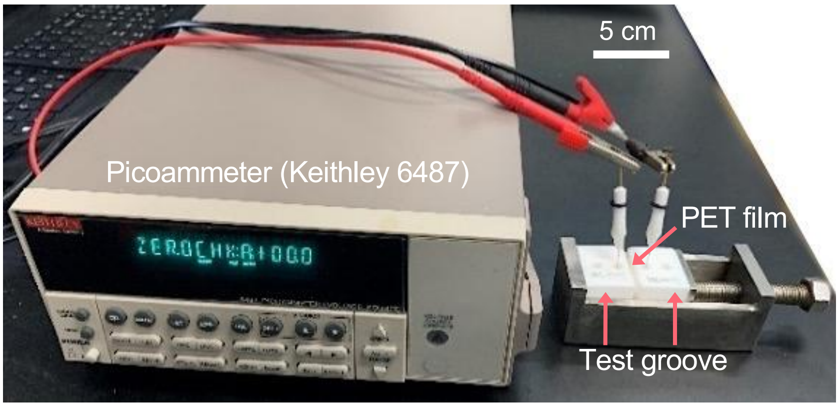

Figure S1. Schematic diagram of ion-track-etching device.

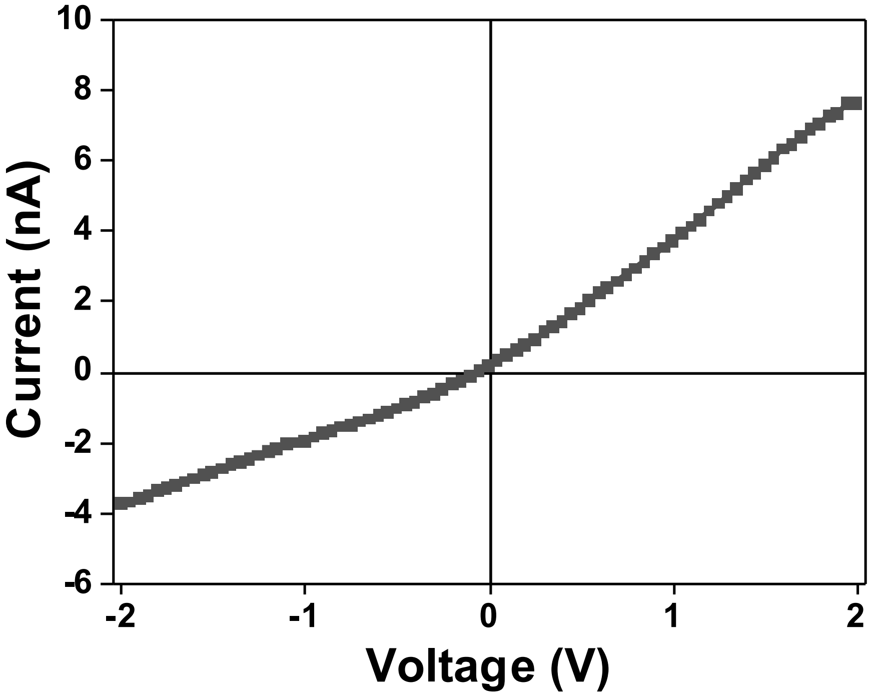

Figure S2. The voltage-current curve of 1M KCl solution test.

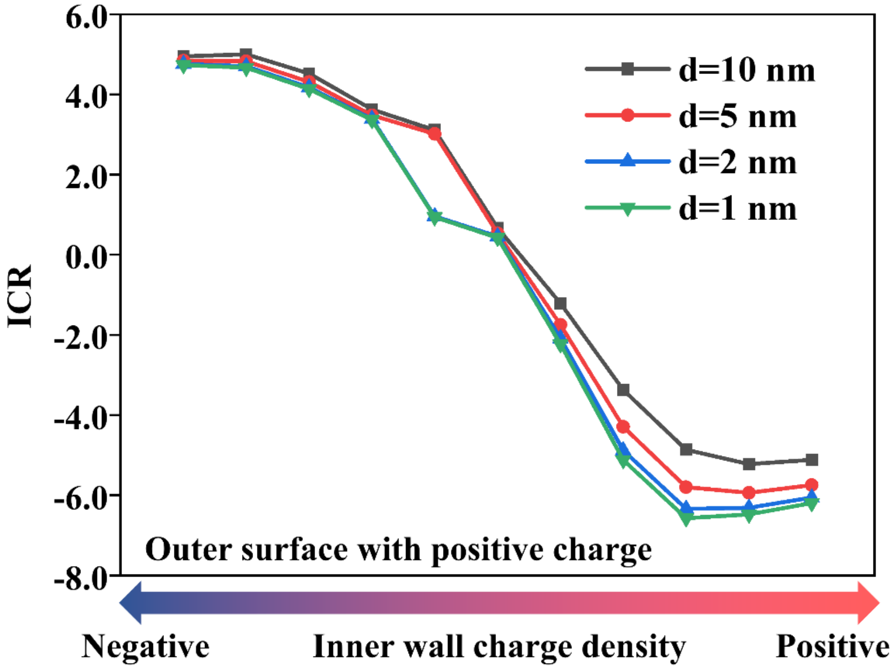

Figure S3. The ICR ratio of the outer surface with positive charge nanochannels with a series of steric hindrance alterations (effecrive aperture, d = 1 nm, 2 nm, 5 nm, 10 nm).

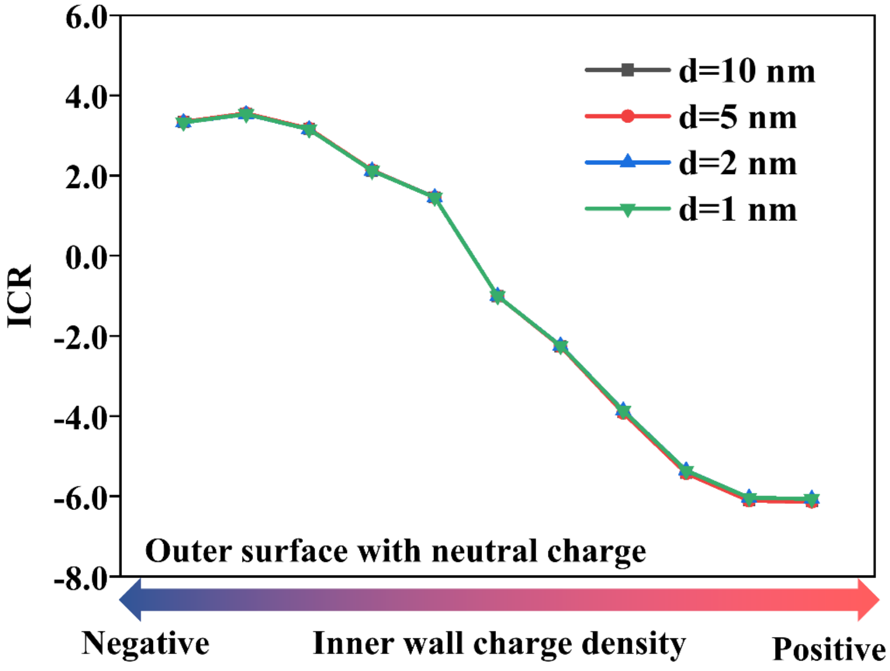

Figure S4. The ICR ratio of the outer surface with neutral charge nanochannels with a series of steric hindrance alterations (effecrive aperture, d = 1 nm, 2 nm, 5 nm, 10 nm).

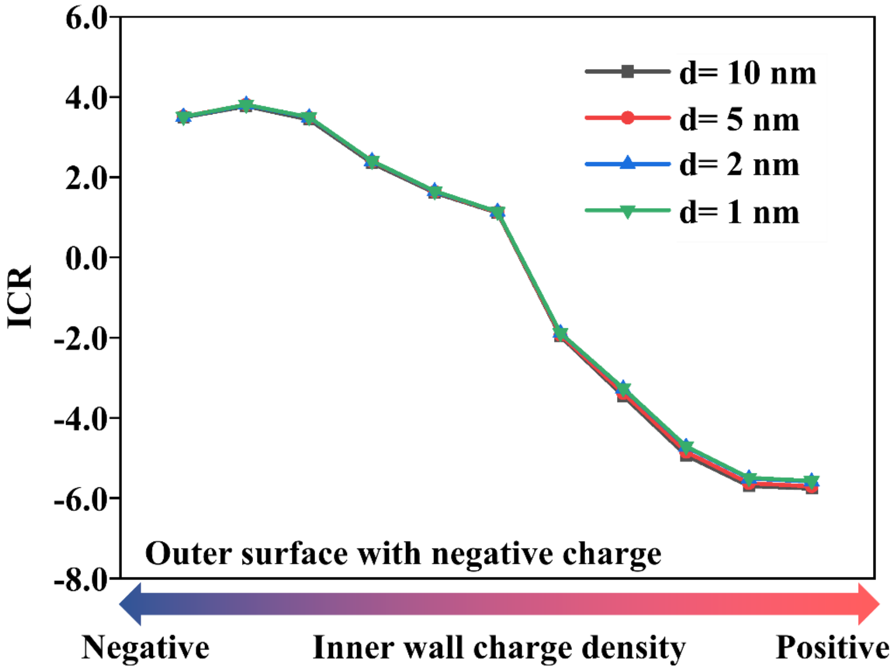

Figure S5. The ICR ratio of the outer surface with negative charge nanochannels with a series of steric hindrance alterations (effecrive aperture, d = 1 nm, 2 nm, 5 nm, 10 nm).
